# Validation and inter-rater reliability of a three item falls risk screening tool

**DOI:** 10.1186/s12877-017-0669-z

**Published:** 2017-11-23

**Authors:** Catherine Maree Said, Leonid Churilov, Kathryn Shaw

**Affiliations:** 1 Physiotherapy Melbourne School of Health Sciences, The University of Melbourne, Level 7, Alan Gilbert Building, 161 Barry St, Parkville, VIC 3010 Australia; 2 Department of Physiotherapy, Austin Health, Heidelberg, Australia; 3 Statistics and Decision Analysis Academic Platform, The Florey Institute of Neuroscience & Mental Health, Heidelberg, VIC 3084 Australia

**Keywords:** Accidental falls, Hospitalization, Nursing assessment, Screening, Validation study

## Abstract

**Background:**

Falls screening tools are routinely used in hospital settings and the psychometric properties of tools should be examined in the setting in which they are used. The aim of this study was to explore the concurrent and predictive validity of the Austin Health Falls Risk Screening Tool (AHFRST), compared with The Northern Hospital Modified St Thomas’s Risk Assessment Tool (TNH-STRATIFY), and the inter-rater reliability of the AHFRST.

**Methods:**

A research physiotherapist used the AHFRST and TNH-STRATIFY to classify 130 participants admitted to Austin Health (five acute wards, *n* = 115 two subacute wards *n* = 15; median length of stay 6 days IQR 3–12) as ‘High’ or ‘Low’ falls risk. The AHFRST was also completed by nursing staff on patient admission. Falls data was collected from the hospital incident reporting system.

**Results:**

Six falls occurred during the study period (fall rate of 4.6 falls per 1000 bed days). There was substantial agreement between the AHFRST and the TNH-STRATIFY (Kappa = 0.68, 95% CI 0.52–0.78). Both tools had poor predictive validity, with low specificity (AHFRST 46.0%, 95% CI 37.0–55.1; TNH-STRATIFY 34.7%, 95% CI 26.4–43.7) and positive predictive values (AHFRST 5.6%, 95% CI 1.6–13.8; TNH-STRATIFY 6.9%, 95% CI 2.6–14.4). The AHFRST showed moderate inter-rater reliability (Kappa = 0.54, 95% CI = 0.36–0.67, *p* < 0.001) although 18 patients did not have the AHFRST completed by nursing staff.

**Conclusions:**

There was an acceptable level of agreement between the 3 item AHFRST classification of falls risk and the longer, 9 item TNH-STRATIFY classification. However, both tools demonstrated limited predictive validity in the Austin Health population. The results highlight the importance of evaluating the validity of falls screening tools, and the clinical utility of these tools should be reconsidered.

## Background

Falls are one of the most common adverse events that occur during hospitalization. Falls can result in injury, increased morbidity, longer hospital lengths of stay and higher health care costs [1, 2]. It has been estimated that the current incidence is between 3 and 13 falls per 1000 bed days [3–5], and approximately one in three people who fall in hospital sustain an injury [6]. A recent study showed that people who fell in hospital had a longer length of stay of around 4 days, and the presence of an injury further increased length of stay by a further 4 days [6]. Over recent years there has been an increasing emphasis on reducing falls in hospital. This is reflected in the accreditation standards for Australian Hospitals (National Safety and Quality Health Service Standards), which includes ‘Preventing falls and harm from falls’ as one of the ten Accreditation Standards [7]. One of the requirements of the standard is that patients are screened for risk of a fall. While several tools have been developed, it is important to validate the tool in the setting in which it will be used [8].

Austin Health is a large tertiary hospital with approximately 980 beds providing acute and subacute care over three campuses. To assist compliance with accreditation guidelines, a system for falls risk screening that could be implemented across the health service was required. As nursing staff complete a number of assessments on admission, the falls screening tool needed to be completed quickly, with the expectation this would maximise compliance and minimise documentation burden.

**Fig. 1:**
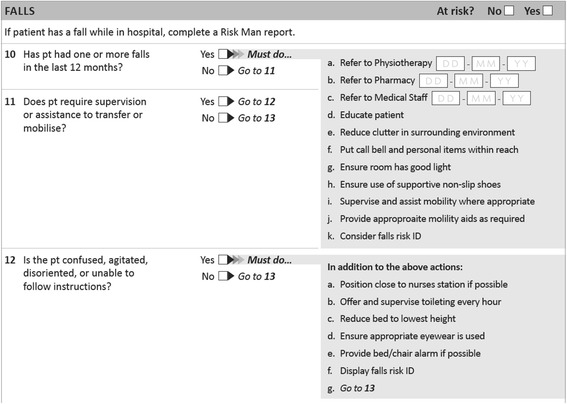
Austin Health Falls Risk Screening Tool

A review of the falls screening or assessment tools at the time identified several tools that had been validated in health services similar to Austin Health, including The Northern Hospital Modified St Thomas’s Risk Assessment Tool (TNH-STRATIFY) and the Western Health Falls Risk Assessment. These tools have multiple items, [9, 10] as do many other falls screening or assessment tools designed for use in the hospital setting [11, 12]. This increases the time required for completion and thus were not considered to be the most appropriate tools for our purpose. Thus a simplified three item standardized screening tool was developed; the Austin Health Falls Risk Screening Tool (AHFRST) which is provided in Fig. 1. This tool was to be administered to all inpatients on admission. However, the validity and reliability of the AHFRST must be evaluated to determine the suitability of this tool to identify potential fallers.

The first aim of this study was to determine the concurrent and predictive validity of the AHFRST in inpatients admitted to Austin Health, by comparing falls classification on the AHFRST with falls classification using TNH-STRATIFY and actual falls. The second aim of this study was to evaluate the inter-rater reliability of the AHFRST by comparing assessments completed by a research physiotherapist with those completed by clinical nursing staff.

## Methods

This was a prospective, cross-sectional study conducted in a large tertiary hospital with approximately 980 beds providing acute and subacute care over three campuses. Ethics approval was gained prior to commencement of the study (Austin Health Human Research Ethics Committee LNNR H2013/05112). The ethics committee approved a waiver of consent for this study, as assessments were low risk and consisted of activities completed during routine clinical care.

### Participants

Participants were recruited from five acute hospital wards (two general medical wards, one general surgical ward, one orthopaedic ward and one neurological ward) and two subacute wards within 48 h of admission. Recruitment from acute wards was on weekdays between 24 July 2013 and 5 August 2013 and recruitment from subacute wards was between 7 August 2013 and 15 August 2013. Consecutive admissions were considered for inclusion. People under the age of 18 years, with an inpatient stay of less than 24 h or people unable to be assessed within 48 h were excluded. People who were cognitively impaired or non-English speaking were included if sufficient information could be obtained from the medical record or if their next of kin could be contacted to provide required information.

Sample size estimation was based on the number of participants required to test the hypothesis of excellent agreement between the AFRAT and the TNH-STRATIFY using these statistics. Under the conventional setting of alpha = 0.05, a sample size of 130 participants would yield 80% statistical power to reliably detect the difference between the agreement of 0.7 (null hypothesis) and 0.8 (alternative hypothesis) using Intraclass Correlation, [13] and the difference between the agreement of 0.6 (null hypothesis) and 0.8 (alternative hypothesis) using Cohen’s Kappa (assuming the proportion of participants at risk of falls is 0.5) [14].

### Procedure

Demographic data including age, gender, admission date, diagnosis, spoken language, and mobility status (pre-morbid mobility and current mobility level) were collected from the medical record or the participant using a standardized data form. A research physiotherapist completed the AHFRST and TNH-STRATIFY with each participant within 48 h of admission. The AHFRST has 3 items (see Fig. 1), with a history of falls in the last 12 months or a mobility impairment requiring supervision/assistance plus cognitive dysfunction indicating high falls risk. The TNH-STRATIFY has been previously validated in an acute clinical setting of a comparable Australian hospital [9]. The TNH-STRATIFY includes nine elements covering a range of fall risk factors that are marked as present or absent, with a score of three or more indicating high falls risk [9]. The order in which the assessments were completed was randomized by flipping a coin. If a participant was cognitively impaired or had insufficient English to participate, information was obtained from the medical record or next of kin, reflecting current clinical practice. If there was insufficient information in the medical record or the next of kin was not contactable within the timeframe, the participant was excluded. Assessments of mobility were undertaken by the research physiotherapist or were determined from a documented physiotherapy assessment within the last 24 h. Confusion and behavioural problems were determined during clinical interaction with the patient and by consulting the clinical notes.

The AHFRST was also completed by clinical staff (usually the admitting nurse) within 24 h of participant admission. These results (Clinical AHFRST) were obtained after the research physiotherapist had completed the assessments, to reduce the risk of bias.

Falls data during the hospital admission were collected from the hospital incident reporting system. A fall was defined as ‘an event which results in a person coming to rest inadvertently on the ground or floor or other lower level’ [15]. If a person had not been discharged 60 days following admission, data was censored at this time point. This approach was taken as it is likely that a person’s clinical condition would change over this extended admission time, thus the initial falls risk screen may no longer be accurate.

### Data analysis

Descriptive statistics including the number of participants classified as high falls risk and number of falls per 1000 bed days was calculated.

To determine the concurrent validity of the AHFRST, the agreement between the AHFRST and the TNH-STRATIFY was examined using three alternative measures of agreement; Cohen’s Kappa Lin’s Concordance Coefficient and Intraclass Correlation Coefficient.

To determine the predictive validity of the AFRAT, sensitivity, specificity, area under the ROC curve, positive predictive values, negative predictive values [16] and the Youden Index [17] were calculated.

To assess the inter-rater reliability of the AFRAT, Cohen’s Kappa, Lin’s Concordance Coefficient and Intraclass Correlation Coefficient were calculated.

## Results

There were 227 people admitted to the wards during the study period; 97 were not included as they were admitted more than 48 h previously (*n* = 43), discharged before assessment (*n* = 31), not available on the ward (*n* = 10) or excluded for other reasons (*n* = 13). A total of 130 participants were recruited to the study; 115 participants from the acute setting and 15 from the rehabilitation setting. The median age was73.3 years (IQR = 54.5–82.6); median length of stay was 6 days (IQR 3–12). Table 1 provides additional demographic characteristics of participants. Three participants had not been discharged 60 days post admission. Six people each fell once during the study period; a rate of 4.6 falls per 1000 bed days.

**Table 1:** Participant Demographics

Characteristic	n (%)
Primary Diagnosis	
Gastrointestinal	22 (16.9%)
Orthopaedic/ Musculoskeletal	32 (24.6%)
Neurological	24 (18.5%)
Cardiac	8 (6.2%)
Respiratory	13 (10.0%)
Fall	5 (3.8%)
Renal	9 (6.9%)
Other	17 (13.1%)
Gender (Male)	53 (41%)
Non English Speaking Background	21 (16.2%)
Cognition	33 (25.4%)
Current Mobility Status	
Assist × 2	14 (10.8%)
Assist × 1	25 (19.2%)
Supervision	26 (20.0%)
Independent	39 (30.0%)
Unable to mobilise	26 (20.0%)

The AHFST classified 55% of participants (*n* = 71 out of 130) at high risk of falls; the TNH-STRATIFY classified 67% (*n* = 87 out of 130) at high risk. There was substantial agreement between the AFRAT and TNH-STRATIFY (Kappa = 0.682, 95% CI = 0.53–0.79, *p* < 0.001). Lin’s concordance and ICC also demonstrated good agreement between the two tools (rho = 0.68, 95% CI =0.59–0.77, *p* < .001; ICC = 0.68, 95% CI = 0.58–0.76).

Details of the predictive validity of the two tests are presented in Table 2. The TNH- Stratify had better sensitivity than the AHFRST, but both tools had poor specificity. Both tools had low positive predictive value, but high negative predictive values. Comparison of the area under the ROC curve demonstrated no significant difference between the AHFRST or the TNH-Stratify (*p* = .301).

**Table 2:** Predictive accuracy of the AHFRST and the TNH-STRATIFY

	AHFRST	TNH-STRATIFY
High Risk (%)	55%	67%
Sensitivity (95% CI)	66.7% (22.3–95.7)	100.0% (54.1–100.0)
Specificity (95% CI)	46.0% (37.0–55.1)	34.7% (26.4–43.7)
AROC^a^	.563 (.352–.774)	.673 (.631–.715)
Positive Predictive Value	5.6% (1.6–13.8)	6.9% (2.6–14.4)
Negative Predictive Value	96.6% (88.3–99.6)	100.0% (91.8–100.0)
Youden Index	0.126 (−0.268–0.485)	0.347 (0.258–0.429)

^a^Area under the ROC curve

The AHFRST was not completed by clinical staff for 14% of participants (*n* = 18 out of 130), thus agreement between classification by the research physiotherapist and clinical staff could only be calculated for 112 participants. There was moderate agreement between the research AHFRST and the clinical AHFRST (Kappa = 0.54, 95% CI = 0.36–0.67, *p* < 0.001). This was confirmed by Lin’s concordance and ICC (rho = 0.54, 95% CI = 0.41–0.67, *p* < .001; ICC = 0.54, 95% CI = 0.39–0.66).

## Discussion

There was good agreement between falls risk classification using the AHFRST and the TNH-STRATIFY; participants identified as ‘high falls risk’ on TNH- STRATIFY were likely to be identified as ‘high falls risk’ on the 3 item AHFRST. However, results indicate that both tools demonstrated poor predictive validity. While the TNH- STRATIFY had better sensitivity than the AHFRST, indicating it was better at identifying participants who fell, both tools had low specificity, indicating neither tool was able to identify those who did not fall. Both tools had low positive predictive values, indicating that only a small proportion of participants classified as ‘high falls risk’ actually fell. It has been recognised that many falls screening tools have low positive predictive values [18]. While the study was not powered to compare the predictive validity of both tools, results indicate no difference in the ability of the two tests to predict falls.

The predictive validity of the TNH-STRATIFY was comparable to the predictive validity of other fall risk screening tools, as indicated by the Youden Index [19]. The previous validation of the TNH-STRATIFY [9] reported a higher Youden Index (0.44; 95% CI 0.32–0.56) and higher positive predictive value (0.23; 95% CI 0.16–0.27) compared with this study. This may be in part due to the higher proportion of participants identified as ‘High Risk’ in the current study. Only 25.1% of participants in the prior study were classified as ‘High Risk’ [9] compared with the 67% classified as ‘High Risk’ using the TNH-STRATIFY in this study. The mean age of participants in this study was approximately 12 years older compared with the prior study (mean age 61.3, sd = 20.7), which may partly explain why a greater proportion of this cohort were classified as ‘High Risk’.

The results also emphasize discrepancies between tool completion as part of routine clinical practice, compared with more rigorous tool completion undertaken as part of a research study. Notably, approximately one out of every seven participants did not have the AHFRST completed by clinical staff. This is of particular concern, given one of the main drivers for developing this three item tool was to maximize compliance. While reasons for non-compliance with tool completion were not explored, this is an important issue which should be further considered. When the AHFRST was completed by clinical staff, there was only moderate agreement with the AHFRST when completed by a researcher. While we did not calculate the predictive validity of the AHFRST when completed by clinical staff, it is likely that the validity of the tool is even lower when completed as part of usual clinical care.

This study highlights the importance of establishing validity of falls screening tools in the local environment in which they are to be clinically utilised. Results are in keeping with previous studies that have shown reduced validity when falls screening or assessment tools are examined in a different hospital or population [8–10]. The clinical value of continuing to use tools with low predictive validity should also be questioned, particularly since the validity is likely to be even lower in routine clinical practice. Both the AHFRST and the TNH –Stratify identified over 50% of participants at ‘high risk’. Over classification of people at ‘high risk’ may mean that people at highest risk may not be appropriately managed. Many fall prevention strategies, such as ensuring a call bell is within reach, adequate lighting and reducing clutter utilise minimal resources and should be considered routine clinical care. However, other strategies are more resource intensive or have practical implications and cannot be delivered to a high proportion of the hospital population. For example, review by other health professionals, positioning close to the nurses’ station or individualised education programs [20, 21] are strategies that may be limited by resources or practicality. Current screening tools do not help guide delivery of these interventions.

There is also a need to consider the resources spent on screening and assessing falls risk, particularly in the acute setting where people are typically in for short periods of time and can have significant changes in medical status. Clinical staff are required to complete a substantial amount of documentation, and there are concerns that this burden may reduce the time spent providing direct patient care [22]. The clinical utility of screening tools is further compromised by non-completion and possible errors in completion when used as part of routine clinical practice, as observed in this study. While advancements such as electronic screening tools may enhance compliance, it is important that potential benefits of generalised screening are balanced against the burdens. Other authors have also queried the importance placed on risk screening in hospital [18]. The revised NICE guidelines from the UK, which were released around the time this study was conceived, state that falls risk prediction tools should not be used in hospital settings [23]. Despite this, many health services continue to rely on falls screening tools. To justify the burden associated with screening, a tool should offer superior predictive validity compared with clinical judgement alone. However, a systematic review of falls risk tools found validity did not differ between ‘clinical judgement’ of falls risk and other developed tools [19]. Clinical judgement has been shown to be influenced by training and experience [24], however the emphasis on use of a validated screening tool may devalue the importance of clinical judgement. Further exploration of the role of clinical judgement in identifying people at high falls risk may be warranted.

One of the limitations of examining predictive validity in this study was that most participants also had falls risk assessed by clinical staff. If a person is identified at high risk of falls, clinical staff are advised to implement a number of interventions to reduce falls risk while the person is an inpatient. These interventions are outlined in Fig. 1 and include strategies such as reducing clutter, providing supervision or assistance with mobility, regular toileting, ensuring the call bell is in reach, referring on to other health professionals, education, falls risk identifiers, positioning close to the nurses station and considering the use of chair/ bed alarms or lo lo beds. While we did not collect data on the presence of interventions to reduce falls, it is likely some interventions would have been implemented for some participants identified at high risk. At the moment there is not strong evidence that these interventions do reduce falls in a mixed acute and subacute hospital population [25]. However, it is possible that staff awareness of falls risk and the presence of fall prevention interventions may have reduced falls in the high risk group. This would lower the sensitivity and positive predictive value of the test. The other challenge when examining the predictive validity of these falls tools in the hospital setting is that participants are only under surveillance for a relatively brief period of time; the median length of stay for participants in this study was only 6 days. Many participants identified at ‘high risk’ may be at risk of falls in the longer term, but they did not fall during their relatively brief hospital stay. This would also negatively impact on the validity of the tool. It is also acknowledged that obtaining falls data from incident reports may have resulted in an underreporting of falls events. Despite these limitations, the findings provide evidence indicating that neither the AHFRST nor the TNH-STRATIFY were valid predictors of falls in this population.

## Conclusion

While the simplified AHFRST had good agreement with the previously validated TNH-STRATIFY, and there was moderate agreement between tool completion by research and clinical staff, both the TNH-STRATIFY and AHFRST had poor predictive validity in this population. Non completion of falls screening tools by clinical staff during routine care, which may reflect screening burden, may further reduce the utility of these tools. The results of this study highlight the importance of evaluating screening tools in the local setting. Furthermore, alternative approaches to falls screening in the hospital setting, including further exploring the role of clinical judgement, should be explored.

## Abbreviations

AHFRST: Austin Health Falls Risk Screening Tool
TNH-STRATIFY: The Northern Hospital Modified St Thomas’s Risk Assessment Tool
